# Glucocorticoid receptor response and glucocorticoid-induced leucine zipper expression in neutrophils of critically ill patients with traumatic and non-traumatic brain injury

**DOI:** 10.3389/fendo.2024.1414785

**Published:** 2024-09-09

**Authors:** N.S. Lotsios, C.S. Vrettou, G. Poupouzas, A. Chalioti, C. Keskinidou, M. Pratikaki, V. Giannopoulou, A. Kotanidou, D.A. Vassiliadi, Ioanna Dimopoulou, A.G. Vassiliou

**Affiliations:** ^1^ First Department of Critical Care Medicine & Pulmonary Services, School of Medicine, National and Kapodistrian University of Athens, Evangelismos Hospital, Athens, Greece; ^2^ Biochemical Department, Evangelismos Hospital, Athens, Greece; ^3^ Department of Endocrinology, Diabetes and Metabolism, National Expertise Center for Rare Endocrine Diseases, Evangelismos Hospital, Athens, Greece

**Keywords:** acute brain injury, corticosteroid response, critical illness, glucocorticoid receptor alpha, glucocorticoid-inducible leucine zipper

## Abstract

**Objective:**

Critically ill patients, including those with brain injuries (BI), are frequently hospitalized in an intensive care unit (ICU). As with other critical states, an adequate stress response is essential for survival. Research on the hypothalamic-pituitary-adrenal gland (HPA) axis function in BI has primarily focused on assessing ACTH and cortisol levels. However, the immunological, metabolic, and hemodynamic effects of glucocorticoids (GCs) are mediated through the glucocorticoid receptor (GCR), a ubiquitously distributed intracellular receptor protein. Data on *GCR-α* expression and its signaling in acute BI injury are lacking.

**Methods:**

We designed a prospective observational study, carried out in one academic multi-disciplinary ICU. Forty-two critically ill patients with acute (BI)were included. These patients suffered from traumatic BI (N= 20), subarachnoid hemorrhage (N= 12), intracranial hemorrhage (N= 7), or ischemic stroke (N= 3). All patients were steroid-free. Twenty-four age and sex-matched healthy controls were used for comparison.

**Results:**

Expression of *GCR-α* and the glucocorticoid-inducible leucine zipper (*GILZ*), serum cortisol, interleukins (IL) 6, 8, 10 and TNF- α, and the BI biomarkers glial fibrillary acidic protein (GFAP) and total Tau were measured on ICU admission (within 48 hours) and 5-7 days from admission. Compared to healthy controls, in the critically ill patients with BI, *GCR-α* mRNA expression was significantly downregulated on admission, and after 5-7 days in the ICU (2.3-fold, p<0.05 and 2.6-fold, p<0.01, respectively). Even though *GCR-α* was downregulated, its downstream gene, *GILZ*, was expressed at the same levels as in normal controls on admission and was significantly upregulated 5-7 days following admission (2-fold, p<0.001). TNF-α levels were undetectable at both time-points. *GCR-α* expression levels inversely correlated with IL-6. The levels of cortisol and the BI biomarkers did not differ between the 2 time-points.

**Conclusions:**

We provide novel evidence on the downregulated expression and upregulated signaling of the ligand-binding and functionally active GCR-α isoform in the polymorphonuclear neutrophils (PMNs) of critically ill patients with BI. The increased *GILZ* expression indicates an increased GC sensitivity in the PMNs of BI critically ill patients.

## 1 Introduction

Severe acute brain injury (BI), representing a significant percentage of intensive care unit (ICU) admissions (1), is primarily caused by traumatic brain injury (TBI), intracerebral hemorrhage (ICH), aneurysmal subarachnoid hemorrhage (aSAH), and acute ischemic stroke (AIS). In acute BI, as in other critical states, an adequate stress response is essential for survival (2). The stress response aims at restoring homeostasis and involves the activation of the hypothalamic–pituitary–adrenal (HPA) axis. This cascade involves the release of corticotropin-releasing hormone (CRH), adrenocorticotropic hormone (ACTH), and cortisol, a vital hormone in regulating stress response, inflammation, and metabolism. Cortisol then inhibits CRH and ACTH secretion via a negative feedback loop (3).

The management of acute brain injuries in the ICU remains a challenge. For many years, corticosteroids have been used in the treatment of patients with brain injuries, as they are believed to lower the intracranial pressure. However, the plethora of randomized controlled clinical and experimental studies exploring corticosteroids in BI have yielded conflicting results (4, 5).

Dysfunction of the HPA axis, mostly in the form of a hyporeactive HPA axis, has been documented in both acute traumatic and non-traumatic brain injuries, with evidence suggesting that these disruptions may persist even after recovery (6–11). BI may affect the HPA axis either by primary injury to the hypothalamic-hypophyseal region or by secondary events, including vascular damage, oedema, vasospasm, and inflammation, which in turn may affect adrenal function (12).

Research on HPA function in BI has primarily focused on assessing ACTH and cortisol levels, whether basal or stimulated. However, it is crucial to acknowledge that the immunological, metabolic, and hemodynamic effects of glucocorticoids (GCs) usually depend on its interaction with the ubiquitously expressed glucocorticoid receptor (GCR) in the cytoplasm. Two human major isoforms of GCR have been identified, with GCR-α being the predominant isoform of the receptor that binds to steroids (13). GCR-β does not bind GCs, it does not transactivate or transrepress target genes, however it acts as a dominant negative inhibitor of GCR-α activity (14). The cortisol-GCR complex directly binds to glucocorticoid responsive elements (GREs) found in GC-target genes, such as the glucocorticoid-inducible leucine zipper (GILZ), which is used as an indicator of glucocorticoid signaling due to its responsiveness to glucocorticoid stimulation (15). Apart from these classical genomic effects via the intracellular GCR, GCs have been found to also exert rapid non-genomic effects by several mechanisms including the activation of a membrane-bound glucocorticoid receptor (mGCR) (16–19).

Dysfunction at the GCR level is equally significant and warrants attention, however no information on GCR signaling is available in BI patients. In a prior study involving unselected critically ill patients, our findings revealed elevated levels of *GCR-α*, yet without concurrent induction of *GILZ* expression on ICU admission, suggesting a state of GC resistance (20). On the other hand, in a subsequent study from our group involving ICU COVID-19 patients, both *GCR-α* and *GILZ* expression levels were increased, indicating a robust activation of the HPA axis (21). These findings suggest that variations in GCR-α expression and function, as evaluated through GILZ, may exist across different causes of critical illness.

The objective of the present study was to examine the expression of *GCR-α* and *GILZ* in critically ill patients with traumatic and non-traumatic BI. This population differs from the general ICU cohorts in that the primary pathology involves direct damage to the brain tissue, which could also result in reduced adrenal stimulation. In this setting, the response of target cells to circulating steroids may be critical. Therefore, we designed a prospective, observational study in which we measured the expression of *GCR-α* and *GILZ* in the polymorphonuclear neutrophils (PMNs) of ICU patients with BI and compared their expression to normal controls. Furthermore, we explored possible correlations of *GCR-α* and *GILZ* expression with BI biomarkers and with cytokines implicated in the GC response during critical illness.

## 2 Materials and methods

### 2.1 Ethical approval

The study was approved by the ‘Evangelismos’ Hospital Research Ethics Committee (467/11-1-2023, Study of ICU patients with brain injury: the role of glucocorticoid receptors and newer brain injury biomarkers on their course, prognosis, and outcomes) and all procedures carried out on patients were in compliance with the Helsinki Declaration. Informed written consent was obtained from all patients’ next-of-kin.

### 2.2 Study design

A prospective, single-center, observational study of 42 ICU patients with BI hospitalized in “Evangelismos” General Hospital (Athens, Greece), over a twelve-month period (January 2023 – December 2023). A control group of 24 healthy subjects, matched for age and sex was also included.

### 2.3 Study population

Prior to enrolment, we screened all consecutive patients for eligibility. Inclusion criteria were new (<48 h) ICU admissions with a diagnosis of BI (including TBI, aSAH, ICH, and AIS). Exclusion criteria were time from initial admission to eligibility assessment >48h, age less than 18 years, pregnancy, diagnosis of brain death, imminent death, estimated length of ICU stay <72 hours, re-admission or transfer from another ICU, contagious diseases (human immunodeficiency virus, hepatitis), oral or inhaled intake of corticosteroids for a period of more than one month prior to ICU admission, and administration of steroids during ICU stay (e.g. hydrocortisone for septic shock). Upon admission in the ICU, the following variables were recorded: age, sex, comorbidities, BI diagnosis, acute physiology and chronic health evaluation (APACHE II) score, sequential organ failure (SOFA) score, vital signs, and laboratory data. The following scales were used for grading the patients’ severity: The Glasgow coma scale (GCS), the Marshal CT classification system (CT score for TBI), the Fisher scale (CT score for aSAH), the World federation of neurological surgeons scale (WFNS) (clinical score for aSAH), the Alberta stroke program early CT score (ASPECTS) (CT score for AIS), and intracerebral blood volume estimation was assessed with the ABC/2 score. Duration of mechanical ventilation and ICU stay along with mortality (28-day and ICU mortality) were also noted. Twenty-four age- and sex-matched healthy blood donors comprised the control group and were used for comparisons.

### 2.4 Blood collection – polymorphonuclear neutrophil isolation

**Figure 1:**
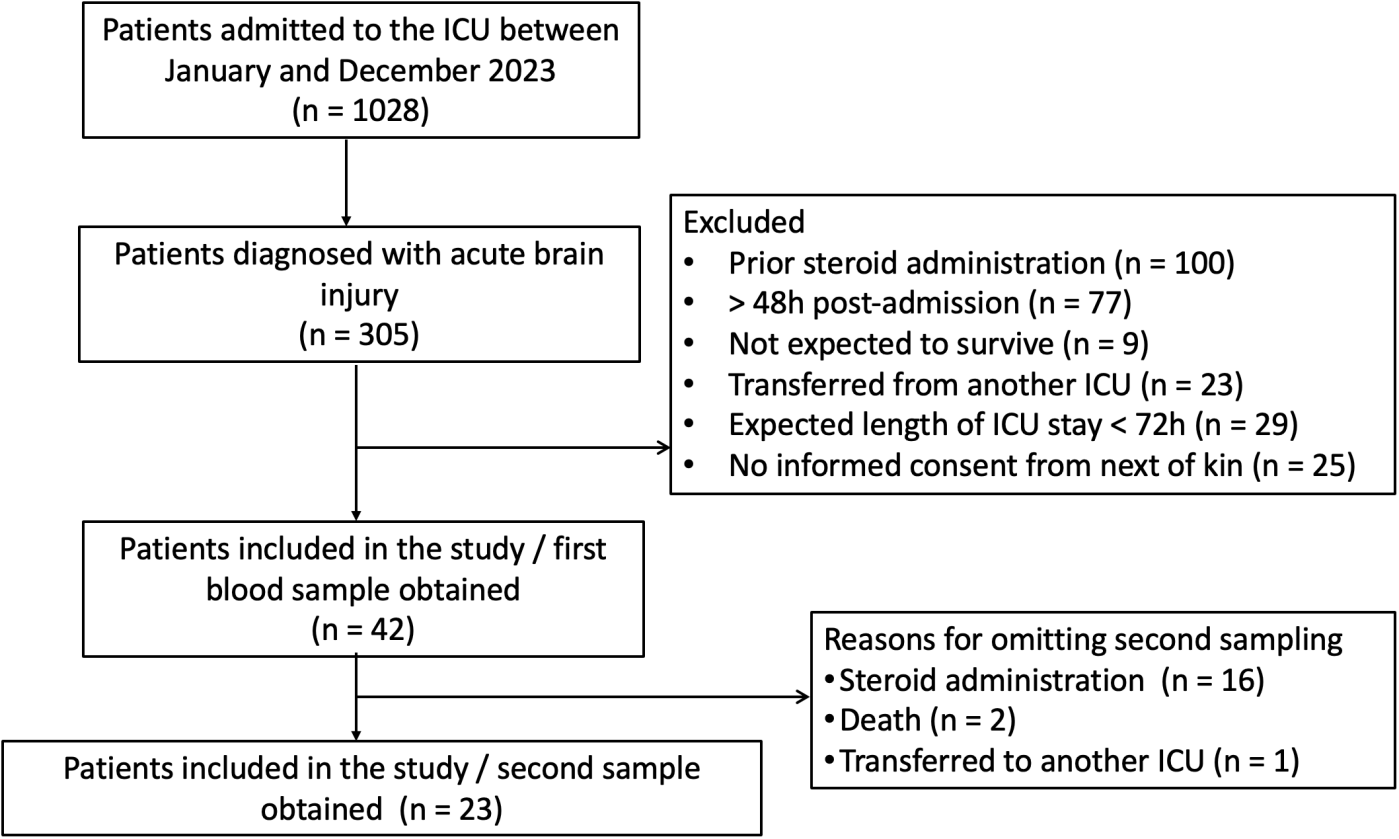
Study flow diagram.

Venous blood samples were drawn from the critically ill BI patients between 08.00 and 08.30 h at two time points: within 48 hours from admission (baseline; 42 patients) and thereafter, at days 5-7 (second time-point, 23 patients). A second blood sample was not obtained when steroid administration due to mainly septic shock, allergic reactions, airway oedema, bronchospasm, transfer to another ICU, or if death occurred. **Figure 1** illustrates the study flow diagram of the enrolment and sampling process.

A single blood sample was also drawn from 24 healthy blood donors between 08.00 and 08.30 h. Blood samples were collected in red top Vacutainers tubes (BD Biosciences, Franklin Lakes, NJ, USA) for serum preparation and blue top Vacutainers citrate tubes (BD Biosciences) for polymorhonuclear neutrophil (PMN) isolation. PMNs were isolated from the peripheral blood, as previously described (14). Briefly, blood was collected in 17% citric acid and allowed to sediment in 3% Dextran/0.9% NaCl for 1 hr. Leukocyte-rich supernatant was collected and any remaining erythrocytes were lysed by hypotonic shock. PMNs were separated by gradient centrifugation on Histopaque (Sigma, Sigma-Aldrich Corp., St Louis, MO, USA) and the isolated PMNs were resuspended in Trizol and stored at -80°C until RNA extraction.

### 2.5 Total RNA extraction and quantitative reverse-transcription PCR

Total RNA was isolated from PMNs, the RNA was reverse transcribed, and a highly sensitive validated quantitative real-time PCR method was used for the quantification of *GAPDH*, *GCR-α*, and *GILZ* mRNAs using specific primers (20, 22). Using the comparative CT method 2^−DDCT^ (23) and the healthy donor samples as a calibrator, the relative quantification of the expression analysis of all blood samples from the critically ill patients was carried out. *GAPDH* expression was used for the normalization of the target gene expression levels between the different samples.

### 2.6 Total cortisol measurement

Total cortisol was determined in the morning blood samples with the Elecsys Cortisol II assay (Roche Diagnostics GmbH, Mannheim, Germany; detection limit 0.054 μg/dl and coefficient of variation 3.1%) with the electrochemiluminescence immunoassay “ECLIA” on a cobas e 411 immunoassay analyzer (Hitachi for Roche Diagnostics International AG, Rotkreuz, Switzerland). The samples were run in duplicates and cortisol levels were expressed in μg/dl. The expected morning normal values of the assay are 4.82‐19.5 μg/dl.

### 2.7 Measurement of cytokines and brain injury biomarkers

The human interleukin (IL) - 6, 8, 10, and tumor necrosis factor (TNF)-α immunoassays from Invitrogen (Thermo Fisher Scientific Inc., Waltham, MA, USA; detection limits IL-6, 0.92 pg/ml; IL-8, 2 pg/ml; IL-10, 2 pg/ml; TNF-α, 5 pg/ml), glial fibrillary acidic protein (GFAP) (OriGene Technologies, Inc., Rockville, MD, USA; detection limit 85 pg/ml), and microtubule-associated tau protein (total Tau) (Wuhan Fine Biotech Co., Ltd.; detection limit 4.688 pg/ml) were used for the measurements, according to the manufacturers’ instructions.

### 2.8 Statistical analysis

Data are presented as individual values, mean ± standard deviation (SD), or median with interquartile range (IQR). Two-group comparisons were performed using the Student’s t-test, the non-parametric Mann-Whitney test, or the Wilcoxon matched-pairs signed rank test, as appropriate. One-way ANOVA for repeated measures and Kruskal-Wallis ANOVA followed by Dunn’s *post hoc* test was used for more than two groups comparisons. Correlations were performed by Spearman’s correlation coefficient. All analyses were performed using the GraphPad Prism 6 statistical program (GraphPad Software, Inc). All p values are two-sided; p< 0.05 were considered significant. Prior to the study a power analysis was performed. Based on a previous study on *GCR-α* expression in PMN cells (12), to detect a 4.7-fold increase of *GCR-α* expression with a Cohen-d effect size of 0.876, an α-error probability <0.05, and a power >80%, it was estimated that a cohort of 42 critically ill patients and a group of 15 matched healthy controls would suffice.

## 3 Results

### 3.1 Characteristics of the study population

Of the 305 patients diagnosed with acute BI, 42 patients were included in the study. The most common cause of exclusion was steroid use prior to ICU admission. Of the 42 patients, 23 patients underwent a second blood sampling. Reasons for not completing the two-step sampling process were administration of steroids (N= 16), death (N= 2), or transfer to another ICU (N= 1). Patients’ demographics, clinical characteristics, and laboratory data on ICU admission, and outcomes are presented in **Table 1**. The BI-related clinical and radiological characteristics are listed in **Table 2**.

**Table 1:** Patient demographics, clinical characteristics, laboratory data, and outcomes of the study population.

Characteristics	
Number of patients, N	42
Age (yr)	55.0 (41.0-66.0)
Sex, N (%)	
Male	24 (57.1)
Female	18 (42.9)
Diagnosis, N (%)	
Traumatic brain injury	20 (47.6)
Aneurysmal acute subarachnoid hemorrhage	12 (28.6)
Acute ischemic stroke	3 (7.1)
Intraparenchymal hemorrhage	7 (16.7)
Glasgow coma scale	6 (3-8)
Comorbidities, N (%)	
Hypertension	14 (33.3)
Hyperlipidemia	11 (26.2)
Diabetes	7 (16.7)
Coronary artery disease	1 (2.4)
Chronic obstructive pulmonary disease	4 (9.5)
Acute physiology and chronic health evaluation II score	17 (12-22)
Sequential organ failure assessment score	9 (7-10)
PaO_2_/FiO_2_ (mm Hg)	322 (223-390)
Intracranial pressure catheter *in situ*, N (%)	
Yes	33 (78.6)
No	9 (21.4)
Type of intracranial pressure monitoring, N (%)	
Intraparenchymal	27 (64.3)
Intraventricular	6 (14.3)
Intracranial hypertension, N (%)	
Yes	12 (28.6)
No	30 (71.4)
Vitals signs	
Heart rate (beats/min)	75 (62-80)
Mean arterial pressure (mm Hg)	85 (80-90)
Temperature (°C)	36.2 (36.0-36.8)
Arterial blood gas analysis	
pH	7.37 (7.33-7.40)
PaO_2_ (mmHg)	129 (95-154)
PaCO_2_ (mmHg)	38 (35-40)
Lactate (mmol/l)	0.9 (0.8-1.5)
Laboratory data	
Hemoglobin (g/dl)	11.15 (9.75-11.92)
White blood cell count (per μl)	12.6 (9.9-16.5) x 10^3^
Platelets (per μl)	201 (179-239) x 10^3^
International normalized ratio	1.01 (1.00-1.10)
Activated partial thromboplastin time (s)	29.7 (26.3-32.3)
Sodium (meq/l)	142 (139-145)
Urea (mg/dl)	25 (19-36)
Creatinine (mg/dl)	0.8 (0.6-1.0)
Glucose (mg/dl)	120 (98-170)
Total bilirubin (mg/dl)	0.53 (0.38-0.64)
C-reactive protein (mg/dl)	3.15 (1.00-9.02)
Fibrinogen (mg/dl)	349 (300-458)
Lactate dehydrogenase (U/l)	221 (171-286)
Mechanical ventilation, N (%)	37 (88.1)
ICU mortality, N (%)	8 (19.0)
28-day mortality, N (%)	7 (16.7)

Data are expressed as percentages of total related variable (%) and median (IQR) for skewed data. Measurements were made on admission to the intensive care unit.

**Table 2:** Clinical and radiological characteristics of the study population on admission to the intensive care unit.

Characteristic	
Marshal CT classification^a^, N (%)	
Diffuse injury I	1 (5.0)
Diffuse injury II	13 (65.0)
Diffuse injury III (swelling)	2 (10.0)
Diffuse injury IV (shift)	1 (5.0)
Evacuated mass lesion V	1 (5.0)
Non-evacuated mass lesion VI	2 (10.0)
WFNS classification^b^, N (%)	
1	1 (8.3)
2	2 (16.7)
3	1 (8.3)
4	1 (8.3)
5	7 (58.4)
Modified Fisher classification^b^, N (%)	
1	0 (0.0)
2	2 (16.7)
3	4 (33.3)
4	6 (50.0)
Intracerebral blood volume estimated with the ABC/2 score^c^, N (%)	
≥300 cc	4 (57.1)
<300 cc	3 (42.9)
Alberta stroke program early CT score (ASPECTS)^d^, N (%)	
≤7	2 (66.7)
>7	1 (33.3)

Data are expressed as percentages of total related variable (%). ^a^assessed in patients with traumatic brain injury (N= 20); ^b^assessed in patients with aneurysmal subarachnoid hemorrhage (N= 12); ^c^assessed in patients with intraparenchymal brain hemorrhage (N= 7); ^d^assessed in patients with acute ischemic stroke (N= 3).

### 3.2 *GCR-α* expression in critically ill patients with brain injury

**Figure 2:**
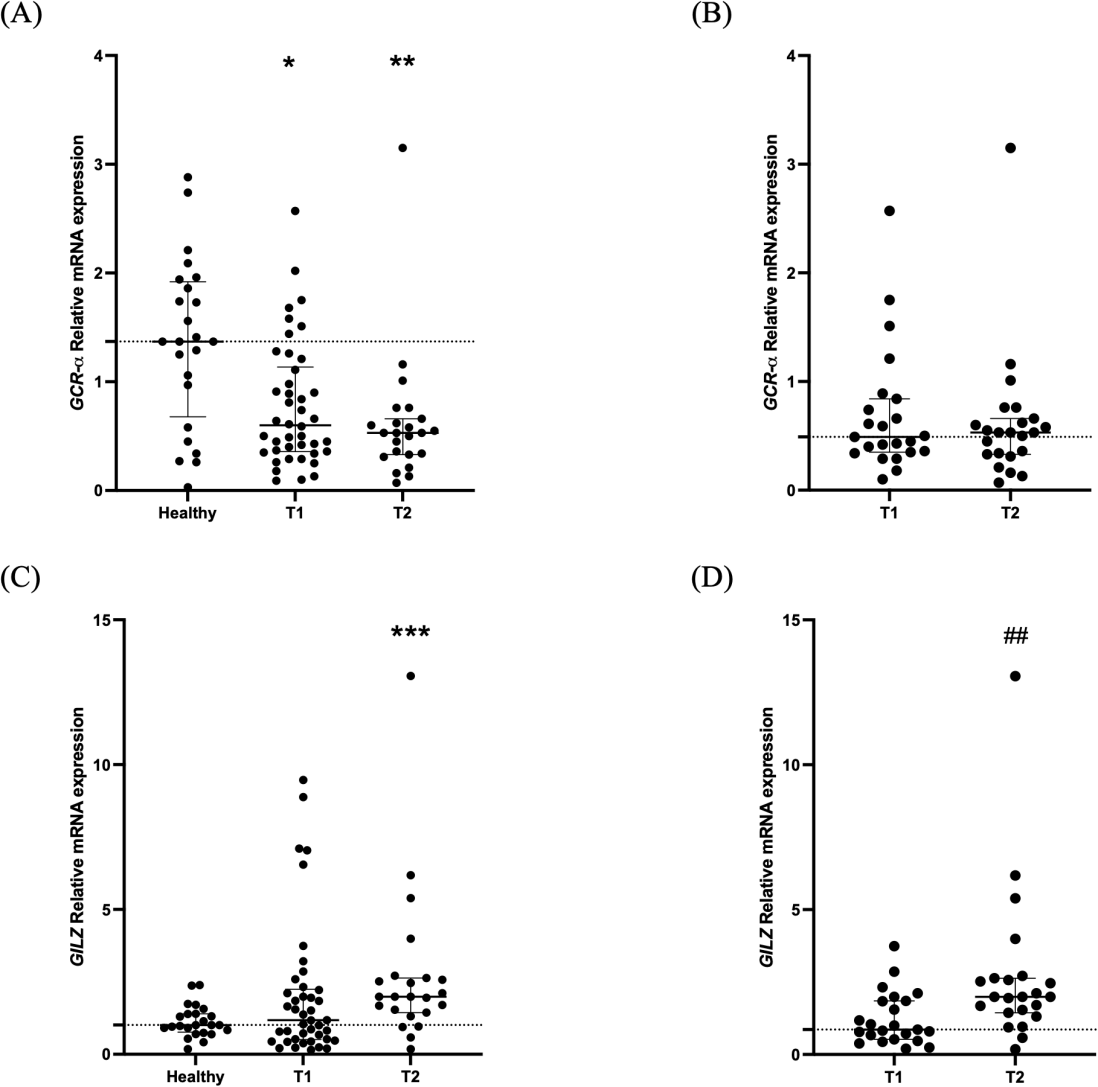
*GCR-α* expression and signaling in critically ill patients with brain injury. Distribution of *GCR-α* expression **(A)**, and *GILZ* expression **(C)** in 42 critically ill patients with brain injury on ICU admission (within 48 hours-T1, N= 42) and 5-7 days after (T2, N= 23). Distribution of *GCR-α* expression **(B)** and *GILZ* expression **(D)** in the 23 critically ill brain injury patients with samples at both time-points. Data are presented as scatter plots, indicating the median value and 25^th^ to 75^th^ centiles. Dashed horizontal line depicts the median value of the healthy subjects **(A, C)** or of the brain injury patients at T1 (N= 23; **B**, **D**). **(A**, **C)**, *p< 0.05, ** p< 0.01, and ***p< 0.001 by Mann-Whitney test compared to the healthy controls; **(B**, **D)**, ##p< 0.01 by the Wilcoxon matched-pairs signed rank test compared to T1.

A control group comprised of 24 healthy blood donors, 54.2% male, with a median age of 53 (47–55) years was used for the relative quantification of the expression analysis of the critically ill patients. Compared to healthy controls, critically ill patients with BI had decreased *GCR-α* expression on ICU admission [0.60 (0.36-1.14); **Figure 2A**; p= 0.014]. Likewise, during ICU stay, on day 5-7, the decreased expression was maintained [0.53 (0.32-0.71); **Figure 2A**; p= 0.0018)]. For the 23 patients who had two blood draws, we also used repeated measures analysis to determine whether expression changed between time points. We found no differences between the two time-points [0.49 (0.36-0.79) vs 0.53 (0.32-0.71); **Figure 2B**; p> 0.05].

The patients were then assigned to 2 subgroups based on diagnosis; patients with TBI (N= 20) and patients with non-traumatic BI (NTBI-ICH, aSAH, and AIS, N= 22). *GCR-a* expression was decreased at both time-points compared to the healthy controls in the TBI subgroup [T1, 0.53 (0.35-0.90) and T2 (N=12), 0.54 (0.17-0.62), p< 0.01]. In the NTBI subgroup, there was a tendency for lower expression at both time-points [T1, 0.62 (0.37-1.32), p= 0.051 and T2 (N= 11), 0.45 (0.34-0.89), p= 0.069]. When the two subgroups were compared against each other, no differences in *GCR-α* expression were found in either time-point (all p> 0.05).

Lastly, when the patients were divided in survivors and non-survivors according to 28-day mortality, *GCR-α* expression on admission was 0.60 (0.36-0.93) in the patients who survived vs 0.86 (0.29-1.95) in the non-survivors (p= 0.41).

### 3.3 *GCR-α* signaling/*GILZ* expression in critically ill patients with brain injury

Compared to healthy controls, critically ill patients had a comparable mRNA expression of the GC-inducible gene *GILZ* on ICU admission [1.18 (0.51-2.25); **Figure 2B**; p= 0.55). On day 5-7, *GILZ* levels increased [1.99 (1.38-2.67; **Figure 2C**; p= 0.0007). As in *GCR-α*, we also compared *GILZ* expression between the two time-points for the 23 patients who had two blood samples. We found an increase in the second time-point [0.86 (0.50-1.92) vs 1.99 (1.38-2.67); **Figure 2D**; p< 0.01].

*GILZ* expression was similar to healthy controls on ICU admission and increased at the 2^nd^ time-point in both TBI and NTBI subgroups. Specifically, for TBI [0.95 (0.56-2.08), p> 0.05 and 2.05 (1.37-2.69), p< 0.05, respectively]. For NTBI [1.44 (0.44-2.39), p> 0.05 and 1.95 (1.19-3.26, p< 0.05, respectively]. *GILZ* expression did not differ between the two BI subgroups at either time-point (all p> 0.05).

Finally, on admission *GILZ* expression was comparable between 28-day survivors and non-survivors [1.27 (0.51-2.25) vs. 1.11 (0.53-5.69), respectively, p= 0.96].

### 3.4 Cortisol, cytokines, and BI biomarker levels in critically ill patients with brain injury

**Figure 3:**
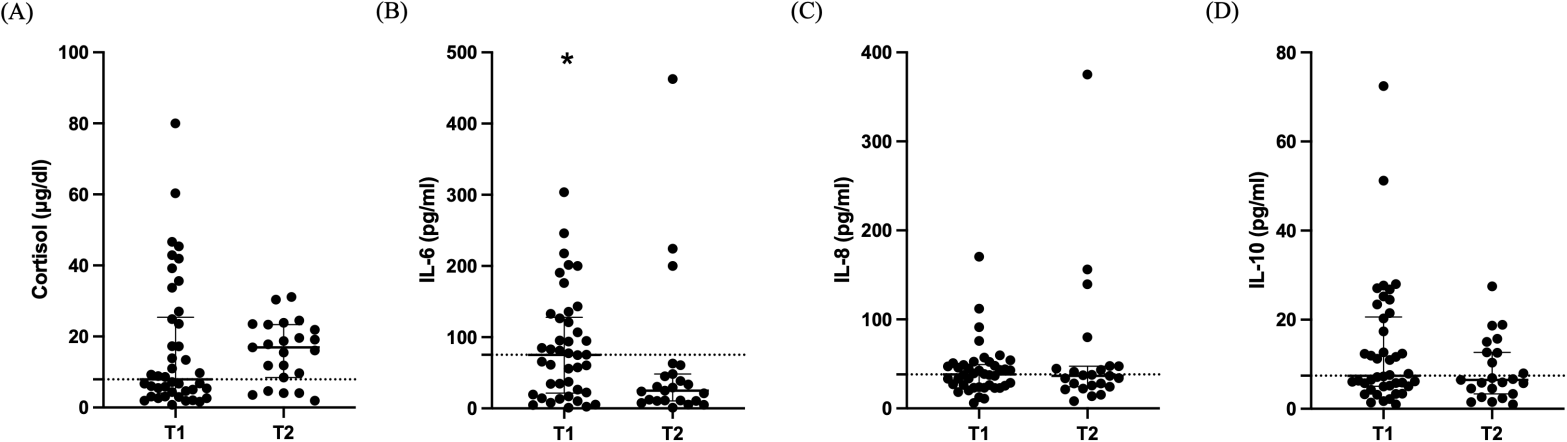
Cortisol and cytokines in critically ill patients with brain injury. Distribution of cortisol **(A)**, IL-6 **(B)**, IL-8 **(C)**, and IL-10 **(D)** in 42 critically ill patients with brain injury on ICU admission (within 48 hours-T1, N= 42) and 5-7 days after (T2, N= 23). Data are presented as scatter plots, indicating the median value and 25^th^ to 75^th^ centiles. Dashed horizontal line depicts the median value of the brain injury patients at T1. *p< 0.05 by Mann-Whitney test.

Cortisol levels did not differ between the two time-points [7.9 (4.1-25.4) μg/dl vs 16.9 (8.5-23.3) μg/dl; **Figure 3A**; p= 0.26]. The change in cytokine levels during the 7-day period is demonstrated in **Figures 3B-D**. IL-6 levels were higher on ICU admission compared to day 5-7 [75.3 (21.6-128.0) pg/ml vs 25.1 (10.5-48.4) pg/ml; **Figure 3B**; p= 0.018]. IL-8 and IL-10 median levels were within normal limits on admission and remained unchanged [38.1 (25.1-49.3) pg/ml vs 36.6 (24.3-47.4) pg/ml; **Figure 3C**; p= 0.74] and [7.4 (5.0-20.6) pg/ml vs 6.5 (3.4-12.7) pg/ml; **Figure 3D**; p= 0.26]. TNF-α levels were undetectable on ICU admission and during ICU stay.

**Figure 4:**
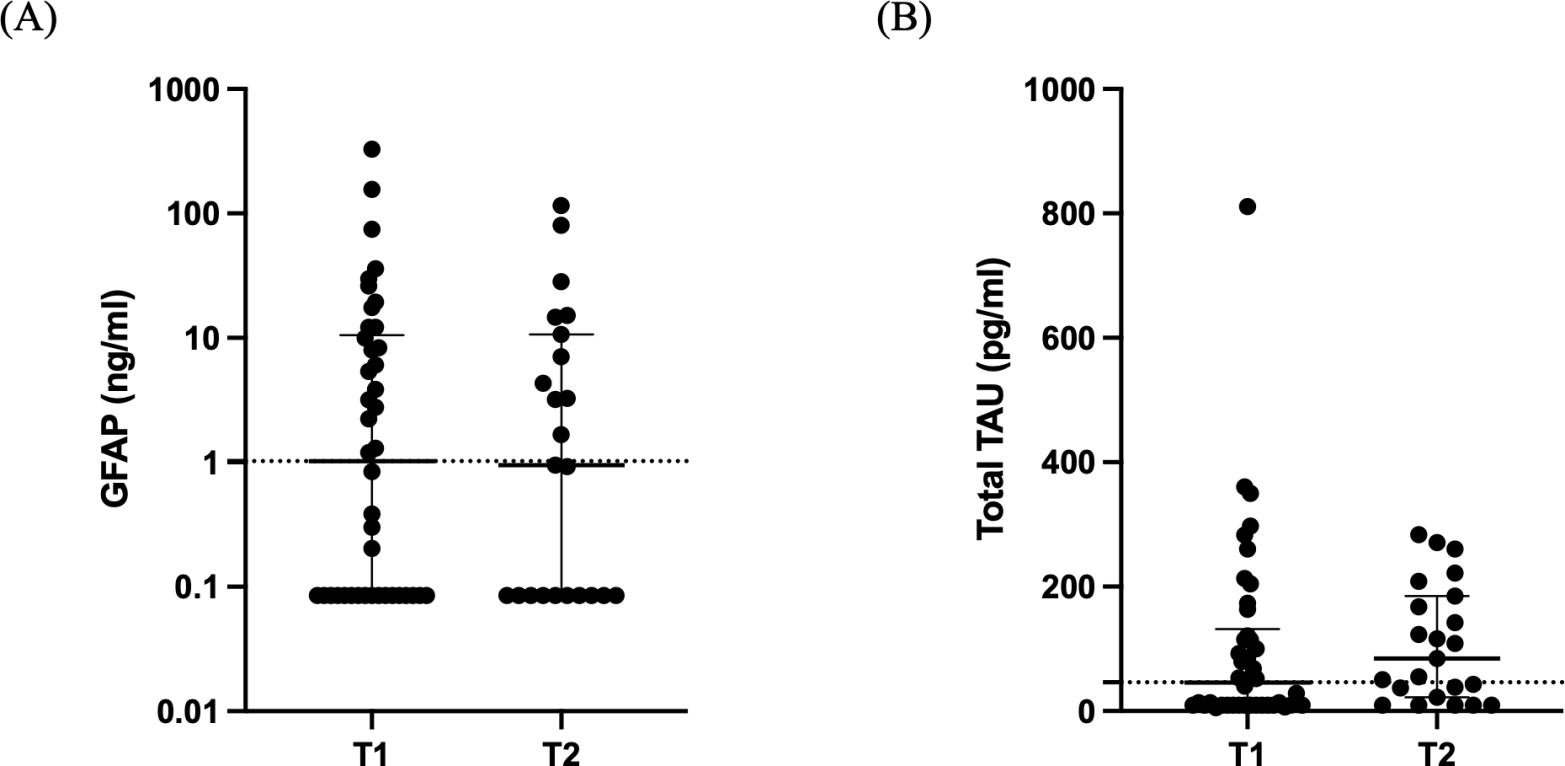
Brain injury biomarkers. Distribution of GFAP **(A)** and total Tau **(B)** in 42 critically ill patients with brain injury on ICU admission (within 48 hours-T1, N= 42) and 5-7 days after (T2, N= 23). Data are presented as scatter plots, indicating the median value and 25^th^ to 75^th^ centiles. Dashed horizontal line depicts the median value of the brain injury patients at T1. GFAP, glial fibrillary acidic protein.

Regarding the BI biomarkers, GFAP levels were high at both time-points [1.022 (0.085-10.51) ng/ml vs 0.951 (0.085-10.66) ng/ml; **Figure 4A**; p= 0.92]. Similarly, total Tau levels were elevated [46.2 (9.4-131.6) pg/ml vs 84.8 (22.1-185.2) pg/ml; **Figure 4B**; p= 0.22].

### 3.5 Correlations of genes’ expression, cortisol, cytokines, and brain injury biomarkers

**Figure 5:**
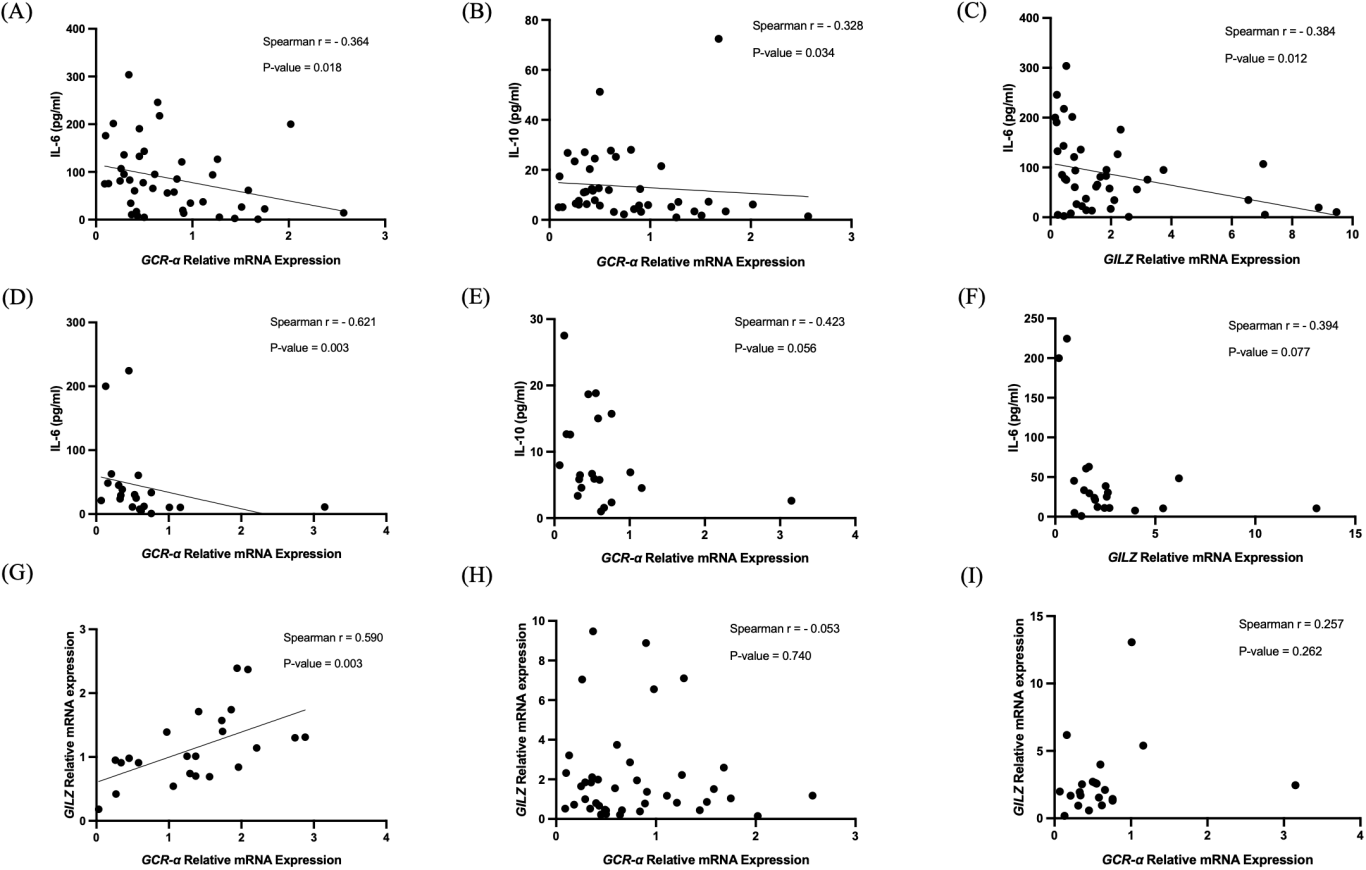
Correlations of *GCR-α* and *GILZ* mRNA expression. Correlation of *GCR-α* mRNA expression levels with IL-6 **(A, D)** and IL-10 **(B, E)** on ICU admission (within 48 hours-T1) and 5-7 days after (T2), correlation of *GILZ* mRNA expression levels with IL-6 **(C, F)** on ICU admission (within 48 hours-T1) and 5-7 days after (T2), and correlations between *GCR-α* and *GILZ* mRNA expression levels in healthy controls **(G)** and in BI patients at both time-points **(H, I)**. Statistical analysis was performed using the Spearman’s correlation coefficient.

On admission, *GCR-α* expression inversely correlated with IL-6 and IL-10 levels (r= -0.36, p= 0.018 and r= -0.33, p= 0.034, respectively), but not with IL-8 (**Figures 5A, B**). Only the inverse correlation with IL-6 remained at the second time-point (r= -0.62, p= 0.003; **Figures 5D, E**). *GILZ* expression inversely correlated with IL-6 levels only (r= -0.38, p= 0.012) on ICU admission (**Figures 5C, F**). Of note, in the healthy patients expression of *GCR-α* and *GILZ* strongly correlated (r= 0.59, p= 0.003; **Figure 5G**), whereas this correlation was not found in the BI patients in either time-point (p= 0.74 and p= 0.26, respectively; **Figures 5H, I**). *GCR-α* and *GILZ* expression did not correlate with cortisol levels, nor with the BI biomarkers GFAP and total Tau. Moreover, no correlations were seen between *GILZ* expression levels and the clinical and radiological characteristics. The statistically significant correlations of gene expression and cytokine levels are depicted in **Figure 5**, while in **Table 3** all correlations between gene expression, cortisol, cytokines, BI biomarkers, and clinical and radiological characteristics are shown.

**Table 3:** Correlations of *GCR-α* and *GILZ* mRNA expression in BI patients with cortisol, cytokines, BI biomarkers, and clinical and radiological characteristics.

Variable	Spearman’s coefficient	p-value
Time-point 1		
*GCR-a* mRNA expression vs		
*GILZ* mRNA expression	-0.053	0.740
IL-6 (pg/ml)	-0.364	0.018*
IL-8 (pg/ml)	-0.074	0.639
IL-10 (pg/ml)	-0.328	0.034*
Cortisol (μg/dl)	-0.063	0.691
Total TAU (pg/ml)	-0.136	0.389
GFAP (ng/ml)	0.036	0.821
*GILZ* mRNA expression vs		
IL-6 (pg/ml)	-0.384	0.012*
IL-8 (pg/ml)	-0.129	0.417
IL-10 (pg/ml)	0.057	0.721
Cortisol (μg/dl)	0.117	0.462
Total TAU (pg/ml)	0.028	0.861
GFAP (ng/ml)	-0.070	0.659
GCS	0.255	0.104
Marshall CT classification	-0.167	0.482
WFNS	0.028	0.937
Time-point 2		
*GCR-a* mRNA expression vs		
*GILZ* mRNA expression	0.257	0.262
IL-6 (pg/ml)	-0.621	0.003*
IL-8 (pg/ml)	-0.160	0.487
IL-10 (pg/ml)	-0.423	0.056
Cortisol (μg/dl)	-0.134	0.561
Total TAU (pg/ml)	0.327	0.148
GFAP (ng/ml)	0.098	0.674
*GILZ* mRNA expression vs		
IL-6 (pg/ml)	-0.394	0.077
IL-8 (pg/ml)	-0.129	0.579
IL-10 (pg/ml)	-0.090	0.699
Cortisol (μg/dl)	0.217	0.345
Total TAU (pg/ml)	0.124	0.592
GFAP (ng/ml)	-0.045	0.845
GCS	0.152	0.511
Marshall CT classification	-0.102	0.757
WFNS	0.278	0.600

GCR-α, glucocorticoid receptor alpha; GCS, Glasgow coma scale; GILZ, glucocorticoid-induced leucine zipper; GFAP, glial fibrillary acidic protein; IL-6, interleukin 6; IL-8, interleukin 8; IL-10, interleukin 10; WFNS, world federation of neurological surgeons’ subarachnoid hemorrhage grading. Correlations were tested with the Spearman’s coefficient. *denotes statistically significant correlations.

## 4 Discussion

In this prospective study we assessed *GCR-α* and *GILZ* expression, as well as serum cortisol, cytokines, and BI biomarker levels in critically ill BI patients. The novel finding was that compared to healthy controls, *GCR-α* expression was low in the PMNs of critically ill BI patients, and cortisol levels were not increased. Nevertheless, GCR signaling occurred, as confirmed by increased expression of *GILZ*, suggesting that our critically ill BI patient cohort does not exhibit GC resistance. Of note, IL-6 levels exhibited an inverse correlation with both *GCR-α* and *GILZ* expression levels, which aligns with the established suppression of expression of the IL-6 gene by GCs through the GCR-α. Finally, although *GCR-a* and *GILZ* expression levels significantly correlated in the healthy controls, this correlation was not seen in BI patients. Overall, our findings suggest that BI patients comprise a distinct subpopulation within the spectrum of critical illness.

While traumatic and non-traumatic BI differ in etiologies, they encompass acquired BI with shared pathophysiology (24). Few studies have assessed GCR expression, number, and affinity, or *GILZ* expression in critically ill patients. These studies have mostly focused on heterogeneous ICU populations, mostly comprising septic patients, and have yielded varied and occasionally conflicting outcomes. While some studies have reported normal GCR count and affinity (25), others have reported evidence of GC resistance, characterized by reduced affinity in peripheral blood mononuclear leukocytes (26), diminished GCR-α expression in T-cells (27), and transient increases in GCR-β expression in mononuclear cells during sepsis (28). Conversely, increased GCR expression and cortisol levels in whole blood during acute-phase sepsis has also been reported (29), implying heightened GC sensitivity. However, elevated GCR-α levels do not necessarily indicate increased GC sensitivity, requiring an assessment of GILZ levels to demonstrate effective GC signaling. In a pilot study it was shown that neutrophils from patients with acute respiratory distress syndrome (ARDS) can express GILZ, whose levels were related to ARDS severity (30).

The concomitant assessment of *GCR-α* and *GILZ* expression has been explored only recently. We have previously found elevated *GCR-α* expression in the neutrophils of critically ill patients of various causes upon ICU admission, with no induction of *GILZ*, indicating ineffective GC signaling (20, 22). In a different ICU cohort with viral sepsis due to COVID-19 we observed increased *GCR-α* and *GILZ* expression in whole blood, suggesting either appropriate HPA axis activation or differential *GILZ* expression among blood cells. The latter is supported by a study of Téblick et al. on a cohort primarily consisting of septic critically ill patients, which demonstrated substantial suppression of *GCR-α* and *GILZ* in neutrophils throughout ICU stay, while monocytes exhibited low/normal *GCR-α* levels alongside increased *GILZ* levels (31). Taken together, it becomes evident that the observed changes in *GCR-α* and *GILZ* expression represent adaptations to the underlying causes of critical illness and may be tissue and cell-specific, and time-dependent.

In BI patients, we initially observed normal *GILZ* expression levels despite low *GCR-α* expression, which increased at a later stage (5-7 days) without a corresponding rise in total cortisol levels. Although increased free cortisol levels might explain these findings, there is limited data on corticosteroid-binding globulins (CBGs) and free cortisol levels in brain injury patients, with one study reporting low free cortisol levels (32). Moreover, CBGs are not expected to decrease as significantly as in sepsis-associated critical illness. Therefore, we believe that the most plausible explanation is the lack of significant GC resistance initially in this cohort, contrasting with critically ill patients of other etiologies, where we observed increased *GCR-α* expression and low *GILZ* expression levels (20, 22), indicative of GC resistance, and further improvement of GC sensitivity as the inflammatory response subsides.

An explanation for the observed absence of notable GC resistance is that our BI patients had a low-grade inflammatory response, as evident by the undetectable levels of TNF-α, which contrasts with previously studied critically ill populations. This distinction is important since the inflammatory response may have an impact on GCR signaling. Pro-inflammatory cytokines can regulate human GCR gene expression. They can either inhibit expression by reshaping the GCR nuclear cofactor profile (33) or increase the steady-state levels of the GCR-β protein isoform over GCR-α, making GCR-β the predominant endogenous receptor isoform, thereby disrupting GC action (34). The increased ratio of the beta isoform to the alpha may in turn increase neutrophil insensitivity towards GCs (35). Hence, data suggest that inflammation itself may contribute to GC resistance (34, 36–38), and thus the absence of significant inflammation may explain the lack of GC resistance in this BI cohort.

The observed inverse relationship between *GILZ* expression levels and IL-6 levels aligns with cortisol’s established role as a suppressor of IL-6, further supporting the notion of an enhanced GC effect in this cohort. Certainly, additional factors such as other hormones or cytokines might also stimulate *GILZ* expression, potentially contributing to the observed upregulation of *GILZ* in our population. Furthermore, we cannot exclude the potential of aldosterone inducing *GILZ* expression through the mineralocorticoid receptor (MR) (39, 40), a receptor also present in the PMNs (41).

In 2005, a large randomized controlled trial on 10,008 adults tested the effect of corticosteroids on death and disability following all head injuries with some Glasgow coma scale (GCS) abnormality. High doses of GCs were related to increased mortality, attributed to immunosuppressive effects, leading to the suggestion that corticosteroids should not be used routinely in the treatment of head injury (42). The results of the CRASH trial may not be applicable uniformly to all brain injury patients and there may be a certain subset of patients who may benefit from corticosteroids. It may be hypothesized that our group of BI patients is not expected to benefit from exogenous administration of GCs, as those administered in the CRASH trial.

Finally, despite the strong correlation between *GCR-α* and *GILZ* expression in healthy subjects, this was notably absent in BI patients at both time-points. Interestingly, in a prior study on COVID-19 sepsis we found a robust correlation between the expression levels of *GILZ* and *GCR-α* (21). The lack of correlation suggests a dissociation between the adrenal response, *GCR* expression, and signaling in BI. This finding warrants further exploration, since it cannot be attributed to BI severity, as indicated by the lack of any significant associations of *GCR-α* and *GILZ* expression levels with serum levels of BI biomarkers, clinical and radiological characteristics, or with the observed patient outcomes.

Amongst the strengths of our study is that, to the best of our knowledge, this is the first study that specifically investigated both *GCR-α* and *GILZ* expression in BI cases, whereas in the previous studies HPA assessment was explored solely through ACTH and cortisol measurements (9, 43). Furthermore, the inclusion of a relatively homogenous BI cohort, with a relatively uniform profile with elevated levels of BI biomarkers, who did not receive exogenous steroid administration and were not confounded by sepsis, constitutes another strength of the present study.

The limitations of our study should also be stated. First, it involved a relatively small number of patients, yet similar to the size of other cohorts examined in prior studies investigating the role of GCR expression levels in critical illness. Second, *GCR-a* and *GILZ* expression measurements were performed in PMNs, rather than monocytes/macrophages, which are considered more relevant circulating target cells for corticosteroid-mediated immune host response in critical illness. Cortisol and cytokine levels were not measured in the healthy blood donors. We only measured GCR-α mRNA expression levels, and not its protein levels. We also only measured total cortisol levels. Free cortisol measurement mirrors better cortisol availability (44), however it is not readily available, and hence total cortisol concentration remains the most suitable marker of adrenal function. Moreover, we have previously shown that in critical illness, tissue cortisol levels correlate to a moderate degree with both total and free cortisol (45). Finally, we did not explore alternative pathways regulating *GILZ* expression, including aldosterone and vasopressin.

## 5 Conclusions

Our data suggest a unique hypothalamic–pituitary–adrenal (HPA) activation pattern in critically ill patients without significant inflammatory responses, such as those with BI. We provide novel evidence on the downregulated expression and upregulated signaling of the ligand-binding and functionally active *GCR-α* isoform in the PMNs of critically ill patients with BI. The increased *GILZ* expression indicates an increased GC sensitivity in the PMNs of BI critically ill patients, suggesting an effective and likely appropriate response to the stress induced by BI, potentially negating the necessity for exogenous steroid administration.

## Data Availability

The raw data supporting the conclusions of this article will be made available by the authors, without undue reservation.
